# Classification of AD and bvFTD using neuropsychological and neuropsychiatric variables: a machine learning study

**DOI:** 10.1002/alz.70782

**Published:** 2025-10-21

**Authors:** Grace J. Goodwin, Jorge Fonseca, Sebastian Mehrzad, Jeffrey L. Cummings, Samantha E. John

**Affiliations:** ^1^ Department of Psychology University of Nevada Las Vegas Nevada USA; ^2^ Department of Brain Health Kirk Kerkorian School of Medicine University of Nevada Las Vegas Nevada USA; ^3^ Department of Computer Science Howard R. Hughes College of Engineering University of Nevada Las Vegas Nevada USA; ^4^ Princeton Neuroscience Institute Princeton University Princeton New Jersey USA; ^5^ Department of Brain Health Chambers‐Grundy Center for Transformative Neuroscience Kirk Kerkorian School of Medicine University of Nevada Las Vegas Nevada USA

**Keywords:** Alzheimer's disease, artificial intelligence, behavioral variant frontotemporal dementia, machine learning, neuropsychiatric symptoms, neuropsychology

## Abstract

**INTRODUCTION:**

Machine learning (ML) is increasingly used for clinical classification of Alzheimer's disease (AD) and related dementias. Prior studies identified useful diagnostic features for AD and behavioral variant frontotemporal dementia (bvFTD), though they often lack pathological verification. We applied ML to classify AD and bvFTD autopsy status using initial visit neuropsychological and neuropsychiatric data.

**METHODS:**

Data from the National Alzheimer's Coordinating Center Uniform Data Set and Neuropathology Data Set were analyzed using logistic regression, support vector machines, random forest, and artificial neural networks to classify autopsy‐confirmed diagnosis based on symptom and cognitive data.

**RESULTS:**

Among 1616 participants (AD = 1498, bvFTD = 118), all algorithms achieved high accuracy (80% to 90%) and discriminatory ability (AUC = 0.89 to 0.95). Apathy, disinhibition, and digit‐symbol substitution were the most important classification features.

**DISCUSSION:**

Findings emphasize the value of specific clinical disease markers to support differential diagnosis of AD and bvFTD.

**Highlights:**

- Four ML algorithms were used for the classification of AD and bvFTD.
- Neuropsychological subtests and neuropsychiatric symptoms were input features.
- Models had high classification accuracy and discrimination.
- We identified important and accessible clinical features for classification.

## 1 BACKGROUND

Early diagnosis of Alzheimer's disease and related dementias (ADRD) facilitates management of symptoms, implementation of interventions, and access to clinical trials.^1^ However, clinical evaluation of ADRD is challenging due to overlapping phenotypes.^1^, ^2^ Assessment of clinical symptoms (e.g., neuropsychological assessment, neuropsychiatric symptom reporting) is integral to diagnosing ADRD across clinical settings, but symptom profiles may not lead to accurate diagnosis, especially at the first clinic visit.

Differential diagnosis of AD and behavioral variant frontotemporal dementia (bvFTD) using clinical data is especially difficult; several clinical features are common in both AD and bvFTD, while others are preferentially, though not exclusively, associated with each syndrome.^3^, ^4^, ^5^, ^6^, ^7^ AD is typically characterized by amnestic symptoms,^8^ and bvFTD is typically characterized by behavioral/personality changes as well as executive dysfunction,^6^, ^7^ though these changes can appear in both conditions.^5^ Studies have shown that misdiagnosed FTD patients resemble an AD clinical profile (e.g., older, fewer neuropsychiatric symptoms), while AD patients with prominent neuropsychiatric symptoms were often misdiagnosed as bvFTD.^9^, ^10^ These diagnostic challenges emphasize the need to identify clinical features that can reliably differentiate AD and bvFTD.

Machine learning (ML) has shown great promise for increasing diagnostic accuracy of ADRD.^11^ ML can identify complex, non‐linear relationships among highly interrelated inputs (e.g., clinical data) that may not be readily observed or detected through traditional statistical approaches.^12^ ML can identify and quantify the importance, or weight, of each input feature in the trained model. These input features can be used to highlight disease features (e.g., symptoms) that are important for accurate distinction of clinical groups.^13^

Research is increasingly using ML to classify AD and bvFTD. Using a Naïve Bayes model, neuropsychological test scores accurately classified 62% of AD and bvFTD cases, while gray matter volumes correctly classified only 51%.^14^ A separate study showed that combining neuropsychological test scores and electroencephalogram features significantly improved classification accuracy, reinforcing the value of integrating multiple data sources for more precise diagnosis.^15^ Additional research using neuropsychological testing as input features found that performance on semantic and phonemic fluency, memory, and attention/working memory were the most important features for classification of AD and bvFTD.^16^ Findings emphasize the potential of ML for advancing differential diagnosis of AD and bvFTD, but further research is needed. Study samples lacked pathological verification, which limits the reliability of findings. Additionally, neuropsychiatric symptoms were not included in the models, despite their prominence in both conditions, particularly bvFTD. Further, testing multiple ML models is critical to increase generalizability and minimize bias.

This study examines whether clinical data collected at initial clinic visit can accurately classify later autopsy diagnosis. We used initial visit data to maximize clinical utility – earlier clinical information can inform prognosis, guide timely intervention, and improve patient outcomes. Although clinical data proximal to death may offer greater diagnostic clarity, our goal is to enhance classification based on the earliest available clinical data. We used the National Alzheimer's Coordinating Center (NACC) neuropathology dataset to select an autopsy‐confirmed sample of AD and FTLD participants. Given that bvFTD is a syndrome rather than a neuropathology, we identified and characterized participants using both the clinically assigned syndrome at initial visit and with neuropathological data. Neuropsychological test data and neuropsychiatric symptom data were selected from the NACC Uniform Data Set (UDS) and used for ML classification of autopsy groups (i.e., autopsy‐confirmed frontotemporal lobar degeneration [FTLD] or autopsy‐confirmed AD). We selected four robust ML models – logistic regression (LR), support vector machines (SVMs), random forest (RF), and artificial neural networks (ANNs) – that are useful for identifying complex, non‐linear patterns in large datasets. Based on previous research,^14^, ^15^, ^16^ we expected story memory and disinhibition to be the most important features for classification. We anticipated that our findings would aid in early, accurate participant classification, addressing diagnostic challenges in ADRD, and improving clinical care.

## 2 METHODS

### 2.1 Participants and sample selection

**RESEARCH IN CONTEXT**

1. **Systematic review**: Differential diagnosis of AD and bvFTD is challenging due to overlapping clinical phenotypes. ML has been shown to accurately classify AD and bvFTD based on clinical data, though samples lack pathological verification. The authors used PubMed to identify research using ML for the classification of AD and bvFTD, and relevant publications are referenced throughout.
2. **Interpretation**: Four ML algorithms accurately classified AD and bvFTD based on initial visit neuropsychiatric and neuropsychological data. Apathy, disinhibition, and the digit‐symbol‐substitution task were consistently important for classification. Findings highlight the utility of ML and specific clinical features for improving differential diagnosis of AD and bvFTD.
3. **Future directions**: While unavailable in our dataset, future research should incorporate comprehensive measures of social functioning for clinical classification of AD and bvFTD. Diverse samples are needed to increase generalizability and clinical translation of findings.

Participants were identified across two NACC datasets, the UDS (all versions), a comprehensive data repository including demographics, neuropsychological, neuropsychiatric, medical, and health history data, and the NACC Neuropathology Data Set, which contains autopsy data for a subset of UDS participants and is one of the largest multisite, multimodal neuropathology databases.^17^ Participant evaluations were completed at Alzheimer's Disease Research Centers (ADRCs) from NACC's inception in 2005 through the September 2024 freeze date. Details on UDS collection methods were described previously.^18^, ^19^, ^20^

**FIGURE 1:**
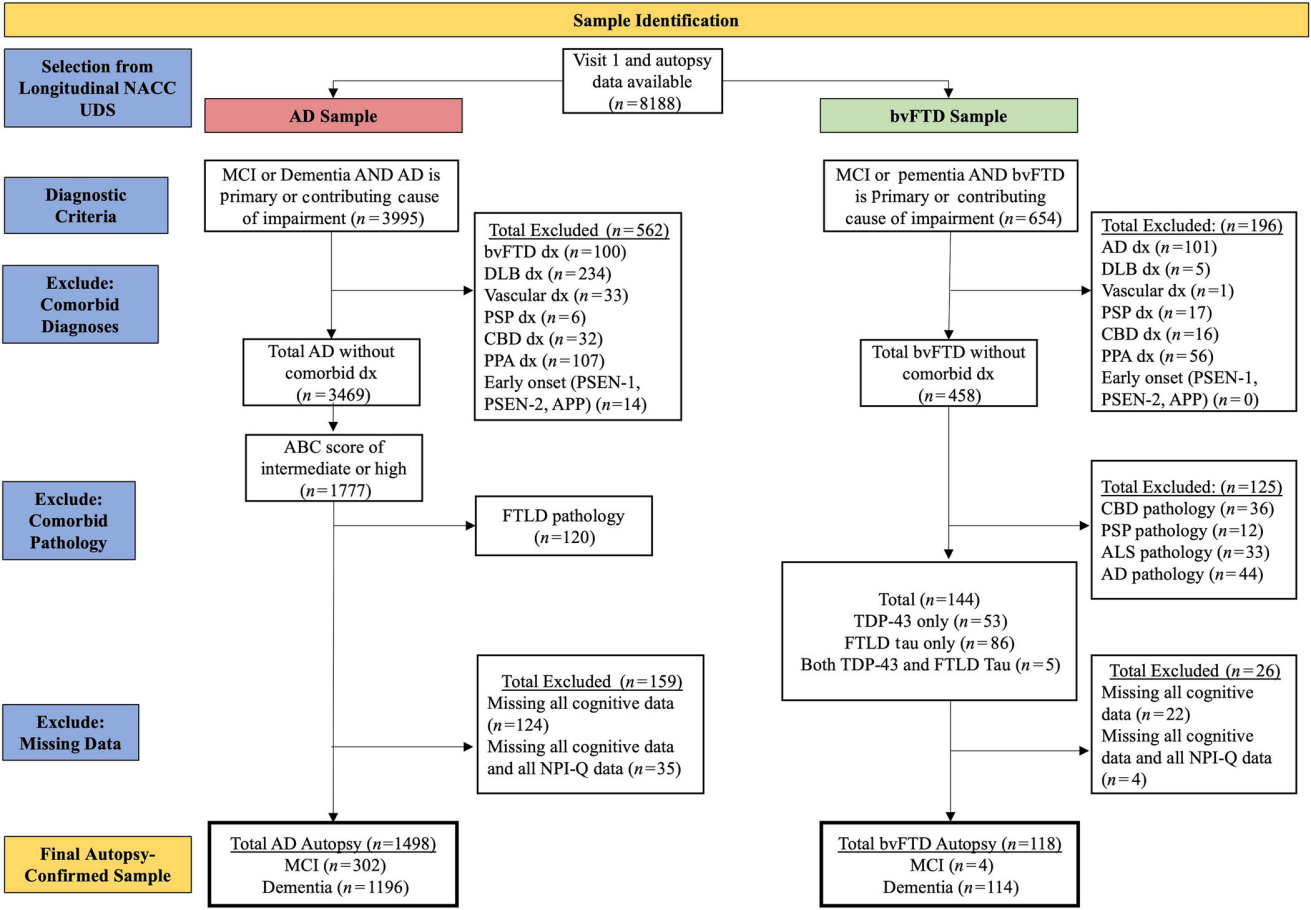
Participant selection diagram. ABC (A: amyloid plaques, B: Braak stage for neurofibrillary degeneration, C: density of neocortical neuritic plaques); AD, Alzheimer's disease; ALS, amyotrophic lateral sclerosis pathology; APP, amyloid precursor protein; bvFTD, behavioral variant frontotemporal dementia; CBD, corticobasal degeneration; DLB, dementia with Lewy bodies; dx, diagnosis; MCI, mild cognitive impairment; NACC, National Alzheimer's Coordinating Center; PPA, primary progressive aphasia; PSEN‐1, presenilin‐1; PSEN‐2, presenilin‐2; PSP, progressive supranuclear palsy; TDP‐43, transactive response DNA‐binding protein 43; UDS, Uniform Data Set.

Participants were identified based on both assigned syndrome diagnosis at initial NACC visit and confirmed autopsy diagnosis (derived from the NACC Neuropathology Data Set) to ensure a well‐characterized and clean sample (*N *= 8188 with initial visit data and autopsy data). Participants with mild cognitive impairment (MCI) or dementia were included in the sample.^19^, ^21^ The following criteria were used for the AD group sample identification: AD was the primary or contributing cause of impairment^8^; no comorbid neurodegenerative syndrome diagnoses at visit 1 (e.g., bvFTD, dementia with Lewy bodies [DLB], primary progressive aphasia [PPA], progressive supranuclear palsy [PSP], corticobasal syndrome [CBS], vascular dementia [VAD]); no evidence of early‐onset AD genetic markers (presenilin‐1 [PSEN‐1], PSEN‐2, amyloid precursor protein [APP]); evidence of AD pathology at autopsy; no evidence of frontotemporal lobar degeneration (FTLD) pathology at autopsy, which includes PSP, corticobasal degeneration (CBD), and amyotrophic lateral sclerosis (ALS).^22^ The following criteria were used for the bvFTD group sample identification: bvFTD was the primary or contributing cause of impairment^7^; no comorbid neurodegenerative syndrome diagnoses at visit 1 (e.g., AD, DLB, PPA, PSP, CBS, VAD); no evidence of early‐onset AD genetic markers (PSEN‐1, PSEN‐2, APP); evidence of FTLD pathology at autopsy; no evidence of comorbid pathology at autopsy (e.g., intermediate or high amyloid/Braak/neurofibrillary tangle [NFT] score; PSP, CBD, ALS). See Figure 1 for the sample identification diagram.

### 2.2 Measures

#### 2.2.1 Participant characteristics

See Table 1 for descriptions of each variable used for sample identification and analyses. Cognitive impairment was defined using the NACC‐derived cognitive status variable for diagnosis (e.g., NACCUDSD = 3 [MCI] or 4 [dementia]), which is determined by a single clinician, a formal consensus panel, or other informal clinician groupings. ADRCs utilize the National Institute on Aging‐Alzheimer's Association (NIA‐AA) guidelines for determining cognitive status. These criteria have been modified over time, and NACC has accordingly referred ADRCs to updated guidelines consistent with emerging research.^21^ The Clinical Dementia Rating (CDR) global staging instrument (0 = *No impairment;* 0.5 = *Questionable impairment*; 1.0 = *Mild impairment*; 2.0 *Moderate impairment*; 3.0 = *Severe impairment*) was also used to characterize participants but was not used for sample selection.^17^, ^20^, ^23^

**TABLE 1:** Description of measures for sample identification and analyses.

Purpose	Domain	Construct/measure	Description
Sample description and selection	Demographics	Demographic	Age, sex, years of education, race, and ethnicity
	Clinical characterization	Cognitive status and primary or contributing cause of observed cognitive impairment	Cognitive status (e.g., MCI vs dementia) and etiologic diagnosis (e.g., primary/contributing cause of impairment) for each patient was determined through a formal process using 2011 NIA‐AA guidelines
	Neuropathology	NIA‐AA AD neuropathologic change	ABC score: Thal phase amyloid plaques, Braak stage for neurofibrillary degeneration, density of neocortical neuritic plaques
		FTLD and TDP‐43 pathology	Neuropathological evidence of FTLD with tau pathology/Pick's disease or TDP‐43 pathology
Input features	Neuropsychiatric functioning	Neuropsychiatric inventory questionnaire	12‐item caregiver report measure of presence and severity of neuropsychiatric symptoms within last month on 4‐point scale
	Cognitive functioning	Category fluency (two categories)	Language functioning
		Trail Making Test Part A	Cognitive processing speed, visuomotor and perceptual scanning, attention
		Trail Making Test Part B	Set shifting, cognitive flexibility
		WAIS‐R Digit Symbol Substitution Test	Cognitive processing speed, visuomotor and perceptual scanning, working memory
		Boston Naming Test/Multilingual Naming Test	Language functioning
		Logical Memory/Craft Story‐21 paraphrase score (delayed recall)	Narrative episodic memory
		Digit span/Number Span Test Forward	Auditory attention
		Digit span/Number Span Test Backward	Working memory/mental tracking

*Notes*: All variables are derived from the NACC Uniform Data Set and Neuropathology datasets.

Abbreviations: ABC, amyloid, Braak, neocortical neuritic plaques; AD, Alzheimer's disease; FTLD, frontotemporal lobar degeneration; MCI, mild cognitive impairment; NIA‐AA, National Institute on Aging‐Alzheimer's Association; TDP‐43, transactive response DNA‐binding protein 43; WAIS‐R, Wechsler Adult Intelligence Scale–Revised.

AD neuropathologic change was determined using NIA‐AA guidelines, in which neuropathologic change is ranked along three parameters and combined into an aggregate “ABC” score: (i.e., A: amyloid plaques, B: Braak NFT stage, C: density of neocortical neuritic plaques recommended by the Consortium to Establish a Registry for Alzheimer's disease [CERAD]).^22^, ^24^, ^25^ Each participant receives an AD neuropathologic change (ADNC) score (*0 = Not AD*, 1 *= Low ADNC*, 2 *= Intermediate ADNC*, 3 *= High ADNC)*. Participants with intermediate or high ADNC were included in the AD autopsy group, as these scores are considered sufficient explanation for AD‐related cognitive change.^24^ Neuropathological change relevant to bvFTD was defined as evidence of FTLD with tau pathology (FTLD‐tau) and/or evidence of FTLD with transactive response DNA‐binding protein 43 (TDP‐43) at autopsy.^26^ A more detailed explanation of NACC neuropathology variables is provided in the NACC Neuropathology Coding Guidebook (https://naccdata.org/).^17^

#### 2.2.2 Neuropsychiatric symptoms

Neuropsychiatric symptoms were measured with the Neuropsychiatric Inventory‐Questionnaire (NPI‐Q), which is a caregiver/informant report of the presence and severity of 12 neuropsychiatric symptoms.^27^ Symptoms include delusions, hallucinations, agitation/aggression, depression/dysphoria, anxiety, elation/euphoria, apathy/indifference, disinhibition, irritability/lability, motor disturbance, nighttime behaviors, and appetite/eating problems. For each symptom, informants first indicate whether the symptom has been present over the last month (0* = symptom not endorsed*, 1* = symptom endorsed*) and then indicate the severity of that symptom (1 = *mild*, 2 = *moderate*, 3 = *severe*). For the present analyses, presence and severity scores were combined into a single variable for each symptom (0 = *symptom not endorsed*, 1 = *mild*, 2 = *moderate*, 3 = *severe*).

#### 2.2.3 Neuropsychological functioning

The NACC neuropsychological test battery (NTB) includes standardized measures of cognitive function that span several domains.^20^, ^28^ Beginning in 2015, proprietary measures used in earlier versions of the NTB were replaced with non‐proprietary tests within the same cognitive domains (Boston Naming Test [BNT] and Multilingual Naming Test [MiNT], Wechsler Memory Scale – Revised [WMS‐R] Logical Memory II and Craft Story‐21, and Digit Span and Number Span). The equipercentile crosswalk table was used to create equivalent scores across versions (e.g., converting non‐proprietary measure scores to proprietary measure equivalent scores).^29^

Language was measured with category fluency (speeded semantic fluency across two semantic categories) ^30^ and BNT/MiNT (confrontation naming).^28^, ^31^ Memory was measured through recall of orally presented story elements (WMS‐R Logical Memory II‐Delayed and Craft Story‐21 Delayed Recall‐paraphrase score).^28^, ^32^ Cognitive processing speed was measured with Trail Making Test Part A (TMT‐A) (rapid, visuomotor, numeric sequencing) and the Wechsler Adult Intelligence Scale – Revised (WAIS‐R) Digit Symbol Substitution Test (DSST; rapid symbol‐digit matching).^33^, ^34^ Executive functioning was measured with Trail Making Test Part B (TMT‐B; rapid, alphanumeric set‐shifting).^34^ Attention and working memory were measured with Digit/Number Span Forward and Backward trials.^19^ Global cognition was estimated with the Montreal Cognitive Assessment (MoCA) ^35^ or the Mini‐Mental State Examination (MMSE),^36^ though these scores were not included in the classification algorithms. Higher scores on TMT‐A and TMT‐B reflect worse performance (i.e., longer time to complete the task), while higher scores on all other measures reflect better performance.

### 2.3 Missing data

Missingness was assessed for NPI‐Q items and neuropsychological subtests.

One hundred and forty‐six participants (124 AD, 22 bvFTD) had missing data on all neuropsychological subtests, and 39 participants (35 AD, four bvFTD) had missing data on all NPI‐Q items and neuropsychological subtests. In general, participants with missing data had severe impairment on the CDR and were unable to complete neuropsychological testing due to a “Cognitive/behavior problem” (code 96 in the NACC UDS codebook). We excluded participants who were missing both NPI‐Q and neuropsychological data, as well as participants who were missing all neuropsychological data, as this level of impairment does not reflect typical functioning within early to moderate stages of disease. We retained participants who were missing only NPI‐Q data (34 AD, four bvFTD), as there was no clinical explanation for their missingness, and values could be imputed.

### 2.4 Statistical analyses

#### 2.4.1 Imputation

Missing data were estimated with Multivariate Imputation by Chained Equations (MICE), which is a flexible method that can handle various variable types (e.g., numeric and factor data). The MICE algorithm imputes missing data based on variable relationships observed within all available data in the dataset. Observed values are regressed on other variables in the imputation model, and missing values are replaced with the predictions from the regression model.^37^ By default, the *mice* package in R uses predictive mean matching for numeric data and has different defaults based on data characteristics. This process is repeated several times, creating multiple “complete” datasets that contain plausible values for missing data points. A total of five imputed datasets were created, and diagnostic plots (e.g., strip plots, convergence plots) were inspected to ensure that imputed values were plausible and followed expected patterns. All ML classification models were run on each of the five imputed datasets to inspect the overall pattern of results and determine consistency of results across each dataset. If models performed similarly across datasets, then the first imputed dataset was selected for model optimization and parameter adjustments.

#### 2.4.2 ML classification models

Four ML classification approaches (LR, SVM, RF, and ANN) were employed to determine the ability of neuropsychiatric symptoms and neuropsychological variables to classify autopsy diagnosis (AD vs bvFTD‐relevant pathology). Classification analyses were performed using the 12 NPI‐Q items and eight neuropsychological subtest scores, and all 20 features were included in each model. Autopsy group was recoded into a binary variable, where bvFTD was the positive class (AD = 0, bvFTD = 1). Complete data were randomly split into a training set (80%) and test set (20%), where the training set was used to predict the classification model, and the testing set was used to determine model performance.

Models were developed using supervised ML algorithms that iteratively adjust internal parameters (weights) during training to minimize classification error (loss function) on labeled data (autopsy group). This iterative learning process enables the model to identify complex patterns within the input features that distinguish groups. To further enhance model performance and generalizability, hyperparameters – settings that govern the learning behavior of the model – were systematically tuned. Where appropriate, oversampling techniques were applied to the training set to address class imbalance (e.g., Synthetic Minority Over‐Sampling Technique [SMOTE] or Adaptive Synthetic Algorithm [ADASYN]). Class weights were adjusted as needed to account for imbalanced groups. Additionally, when indicated, standardization or min‐max normalization was applied to the training set for models that required feature scaling to ensure all input variables are on a comparable scale.^38^ Otherwise, raw data were used for model training and testing. Final model performance was assessed on the independent, non‐oversampled, test dataset. Reported models reflect the best performance in terms of sensitivity, specificity, and overall classification accuracy on unseen data (i.e., the test dataset).

##### 2.4.2.1 Logistic regression

LR is a supervised ML approach for binary classification problems and models the relationship between independent variables (e.g., 20 clinical features) and the probability of an outcome to be in a particular class (e.g., AD or bvFTD).^39^ LR was trained and then tested on the test dataset to determine model performance. The LR models were built using the *scikit‐learn* library in Python.^40^

##### 2.4.2.2 Support vector machines

SVMs are a robust, supervised ML approach for classification problems. SVMs classify data by learning an optimal decision boundary (e.g., a line or hyperplane) that maximally separates groups (AD and bvFTD). By maximizing the margin between support vectors (the data points closest to the decision boundary), SVMs aim to improve generalization to the test dataset.^41^ SVMs can model complex, non‐linear relationships using kernel functions, allowing SVMs to implicitly map input features into higher‐dimensional spaces where a linear separation is more feasible, without explicitly computing the transformation. This makes SVMs particularly powerful when data are not linearly separable in their original feature space. The SVM models were implemented using the *e1071* package in R,^42^ and each model was trained on the 20 neuropsychiatric and neuropsychological features. Both linear and non‐linear (e.g., radial basis function) kernels were tested to assess the presence of linear and non‐linear structures in the data.

##### 2.4.2.3 Random forest

RF ML classification is a supervised, ensemble, tree‐based ML approach often used for classification problems.^43^ RF trains an ensemble of decision trees to perform classification, where each decision tree is trained using random samples of the data and a random selection of features at each step. Each tree makes a prediction (e.g., AD or bvFTD) based on the input data (e.g., 20 features). The algorithm combines predictions of all the trees through majority voting, at which point the final classification is made (e.g., AD or bvFTD).^43^ Feature importance is determined based on the Gini impurity index (or Gini importance), which calculates how well a certain feature can split the data into groups. Features that lead to lower Gini impurity are considered important for classification.^43^ The RF model was built using the *scikit‐learn* library in Python.^40^

##### 2.4.2.4 Artificial neural networks

ANNs are a flexible deep learning approach that can recognize complex, non‐linear interactions among input features to determine an outcome. ANNs loosely simulate the function of synaptic transmission in the human brain, where information is transmitted across layers of interconnected “neurons.” A basic ANN consists of an input layer (e.g., 20 neuropsychological and neuropsychiatric features), one or more hidden layers, and an output layer that produces the final classification (e.g., AD or bvFTD).^44^ Neurons in each layer are connected to the previous and subsequent layers through weighted edges that determine the influence of one neuron on another.^45^ The number of neurons in each layer can vary, allowing the model to detect subtle patterns that might be missed by traditional methods. ANNs are trained iteratively over multiple cycles (or “epochs”) to minimize classification error. Feature importance was determined using the Shapley Additive explanation (SHAP),^46^ which quantifies the contribution of each feature to the model's output. ANNs were built using the *Keras* ^47^ and *TensorFlow* libraries in Python.^48^

#### 2.4.3 Assessing model performance

Several metrics were analyzed for each model to evaluate model performance. Each algorithm was evaluated using accuracy, sensitivity, specificity, positive predictive value (PPV), and negative predictive value (NPV). F1‐score, or the harmonic mean of PPV and sensitivity, was also calculated, as this metric provides a more stable evaluation of model performance when there is class imbalance or imbalanced sample sizes. The F1‐score balances the types of errors and the number of incorrect predictions and provides a better metric for model performance across both classes. This is useful when the classification accuracy of the larger group may influence the overall accuracy score.^49^ Receiver operating characteristic (ROC) curves were estimated for each model, and the areas under the ROC curves (AUC) were calculated to determine the models’ ability to discriminate between the two autopsy groups. The optimal models were determined by these metrics.

## 3 RESULTS

### 3.1 Clinical and demographic characteristics

The final analyzed sample (*N* = 1616; AD group = 1498 [49.8% female], bvFTD group = 118 [33.1% female]) included well‐educated (AD: *M_ed_ = *15.33 [3.14]; bvFTD: *M_ed _= *15.56 [2.72]) participants who predominantly identified as non‐Hispanic white (AD: *N *= 1324 [88.4%]; bvFTD: *N* = 110 [93.2%]). The AD group was significantly older than the bvFTD group (AD: *M_age_ = *72.18 [10.02]; bvFTD: *M_age _= *62.19 [6.88]) (*t*[159.05] = 14.61, *p *< 0.001, *g* = 1.02). The majority of the sample met criteria for dementia (AD dementia: *N *= 1196 [79.8%]; bvFTD dementia: *N *= 114 [96.6%]). See Table 2 for additional sample characteristics.

**TABLE 2:** Participant demographics by autopsy group.

Sample Characteristic	AD	bvFTD
*N*	1498	118
Age (mean [SD])^b^	72.18 (10.02)	62.19 (6.88)
Sex = Female (%)^a^	746 (49.8%)	39 (33.1%)
Education (mean (SD))	15.33 (3.14)	15.56 (2.72)
Ethnicity and race (%)		
Non‐Hispanic White	1324 (88.4%)	110 (93.2%)
Hispanic White	50 (3.3%)	1 (0.8%)
Non‐Hispanic Black	62 (4.1%)	1 (0.8%)
Hispanic Black	2 (0.1%)	0 (0.0%)
Other	60 (4.0%)	6 (5.1%)
Cognitive status at visit (%)		
MCI	302 (20.2%)	4 (3.4%)
Dementia	1196 (79.8%)	114 (96.6%)
CDR global impairment rating (%)		
None (0.0)	5 (0.3%)	1 (0.8%)
Questionable (0.5)	682 (45.5%)	31 (26.3%)
Mild (1.0)	627 (41.9%)	54 (45.8%)
Moderate (2.0)	158 (10.5%)	27 (22.9%)
Severe (3.0)	26 (1.7%)	5 (4.2%)

*Notes*: Impairment ratings derived from the Clinical Dementia Rating Global Impairment score; other ethnoracial group includes those identifying as Asian, American Indian/Alaska Native, Native Hawaiian or Other Pacific Islander, Multiracial, and Unknown.

Abbreviations: AD, Alzheimer's disease; bvFTD, behavioral variant frontotemporal dementia; CDR, Clinical Dementia Rating; MCI, mild cognitive impairment; SD, standard deviation.

^a^ Groups differed at *p *< 0.05.

^b^ Groups differed at *p *< 0.001.

### 3.2 Neuropsychiatric symptom severity

**FIGURE 2:**
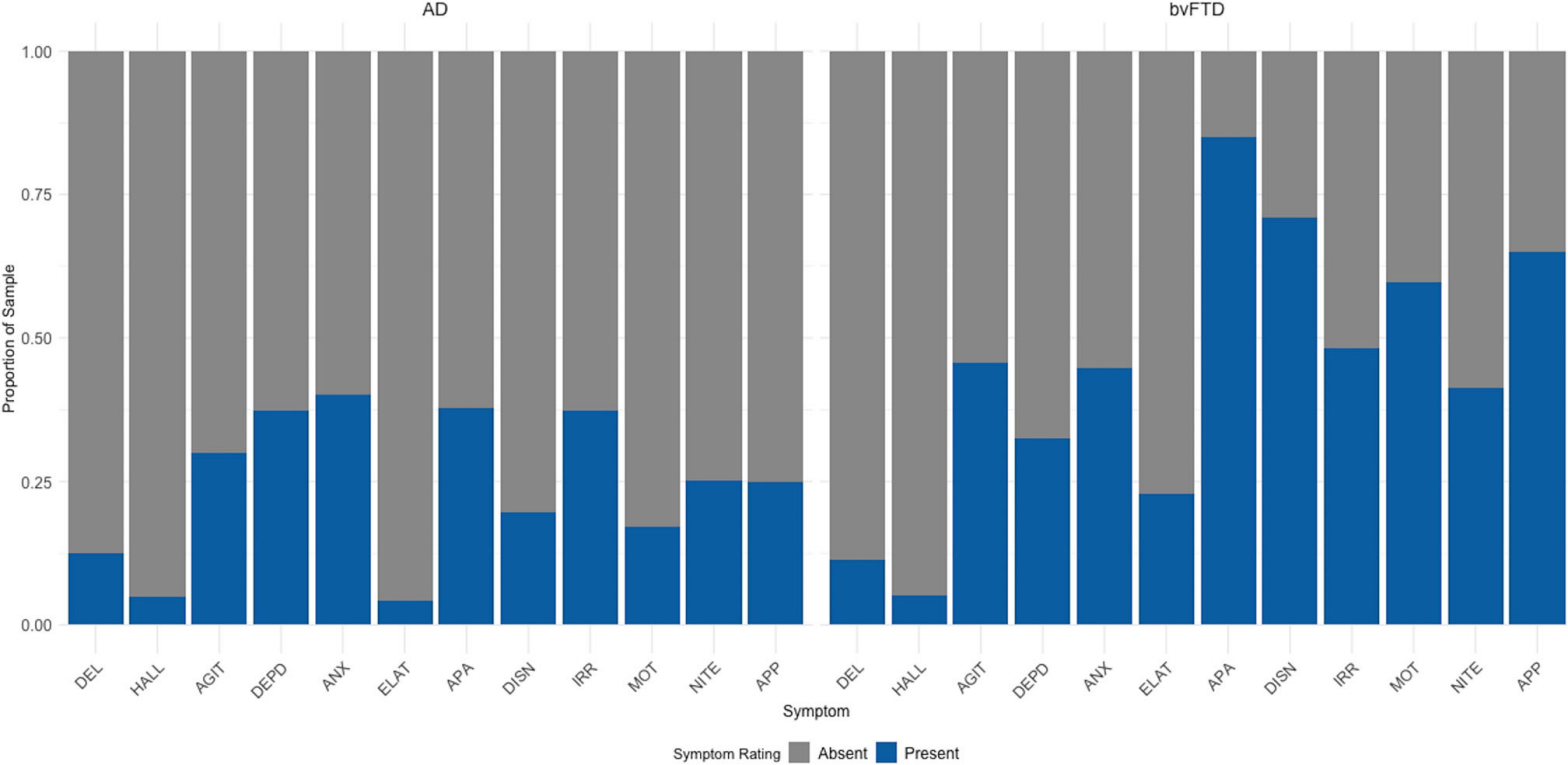
Frequency of endorsed neuropsychiatric symptom items at initial visit. Left panel = AD group, right panel = bvFTD group; AD, Alzheimer's disease; AGIT, agitation/aggression; ANX, anxiety; APA, apathy/indifference; APP, appetite/eating problems; bvFTD, behavioral variant frontotemporal dementia; DEL, delusions; DEPD, depression/dysphoria; DISN, disinhibition; ELAT, elation/euphoria; HALL, hallucinations; IRR, irritability/lability; MOT, motor disturbance; NITE, nighttime behaviors; NPI‐Q, Neuropsychiatric Inventory‐Questionnaire.

Neuropsychiatric severity was generally higher for the bvFTD group (*M *= 9.74 [5.78]; median severity = 10) compared to the AD group (*M *= 4.25 [4.07]; median severity = 3) (*t*[121.92] = 9.94, *p *< 0.001, *g *= 1.30). On average, the AD group reported fewer NPI‐Q items (median number of symptoms endorsed = 2) compared to the bvFTD group (median number of symptoms endorsed = 5). Fisher's exact tests showed that symptom severity differed between groups for all NPI‐Q items, apart from delusions, hallucinations, and depression/dysphoria (Table S1). Anxiety was the most endorsed symptom among AD participants (40.23% endorsed), and apathy/indifference was the most endorsed symptom among bvFTD participants (85.09% endorsed) (Figure 2).

### 3.3 Cognitive performance

The bvFTD group performed better than the AD group on TMT‐A (*M_bvFTD_* = 55.45 [33.27], *M_AD_* = 64.69 [39.62]; *p *= 0.01, *g *= 0.24), TMT‐B (*M_bvFTD_* = 165.77 [95.06], *M_AD_* = 194.61 [89.71]; *p *= 0.02, *g *= 0.32), DSST (*M_bvFTD_* = 32.86 [15.46], *M_AD_* = 28.10 [14.24]; *p *= 0.01, *g *= 0.33), and story memory (*M_bvFTD_* = 4.51 [4.54], *M_AD_* = 2.44 [3.48]; *p *< 0.001, *g *= 0.58). The AD group performed better than the bvFTD group on category fluency (*M_bvFTD_* = 15.93 [9.59], *M_AD_* = 19.05 [8.95]; *p *= 0.002, *g *= 0.35) and naming (*M_bvFTD_* = 19.74 [8.15], *M_AD_* = 21.52 [7.13]; *p *= 0.03, *g *= 0.25). Groups did not differ on digits forward, digits backward, and global cognition (Table 3).

**TABLE 3:** Differences in neuropsychological scores between autopsy groups.

Cognitive Subtest	AD (mean [SD])	bvFTD (mean [SD])	*g*
Trail Making Test Part A	64.69 (39.61)	55.45 (33.27)	0.24^a^
Trail Making Test Part B	194.61 (89.71)	165.77 (95.06)	0.32^a^
DSST^c^	28.09 (14.23)	32.86 (15.46)	0.33^a^
Category fluency	19.05 (8.95)	15.93 (9.59)	0.35^a^
Naming	21.52 (7.13)	19.74 (8.15)	0.25^a^
Story memory	2.44 (3.48)	4.51 (4.54)	0.58^b^
Digits forward	7.01 (2.34)	7.16 (2.45)	0.06
Digits backward	4.50 (2.09)	4.06 (2.71)	0.21
Global cognition	21.79 (5.69)	22.36 (6.19)	0.01

*Notes*: Means, standard deviations, and effect sizes of neuropsychological scores between the AD and bvFTD autopsy groups. Category fluency represents the total score on two semantic categories. Naming, story memory, digits forward, and digits backward were calculated using the NACC equipercentile equating method between test versions within that category (e.g., Naming = Boston Naming Test/Multilingual Naming Test; story memory = WMS‐R Logical Memory II‐Delayed Recall/Craft Story‐21 Recall‐Delayed (paraphrase score); digits forward = Digit Span Forward/Number Span Forward; digits backward = Digit Span Backward/Number Span Backward); global cognition = MoCA/MMSE. Higher scores reflect worse performance on Trail Making Test Part A and Part B. *P* values were calculated using Welch's *t* tests for unequal variances.

Abbreviations: AD, Alzheimer's disease; bvFTD, behavioral variant frontotemporal dementia; DSST, WAIS‐R Digit Symbol Substitution Test; SD, standard deviation; *g*, Hedge's *g* measure of effect size.

^a^ Groups differed at *p* < 0.05.

^b^ Groups differed at *p* < 0.001.

^c^ Denotes important feature in classification models.

### 3.4 ML algorithms

Algorithms were run on each of the five imputed datasets, and classification results were similar across all datasets. As such, we chose to optimize the models for the first imputed dataset and focus on those results below. Several methods were implemented to address class imbalance of the AD (*n *= 1498) and bvFTD (*n *= 118) groups and mitigate the model's tendency to favor the majority class (e.g., AD group). When indicated, class‐weight adjustment scaling and oversampling techniques (e.g., SMOTE or ADASYN) were applied to the training data to address class imbalance. Additionally, the decision threshold was tuned on the test data if needed to improve the correct identification of the minority class (e.g., bvFTD).

#### 3.4.1 Logistic regression

**FIGURE 3:**
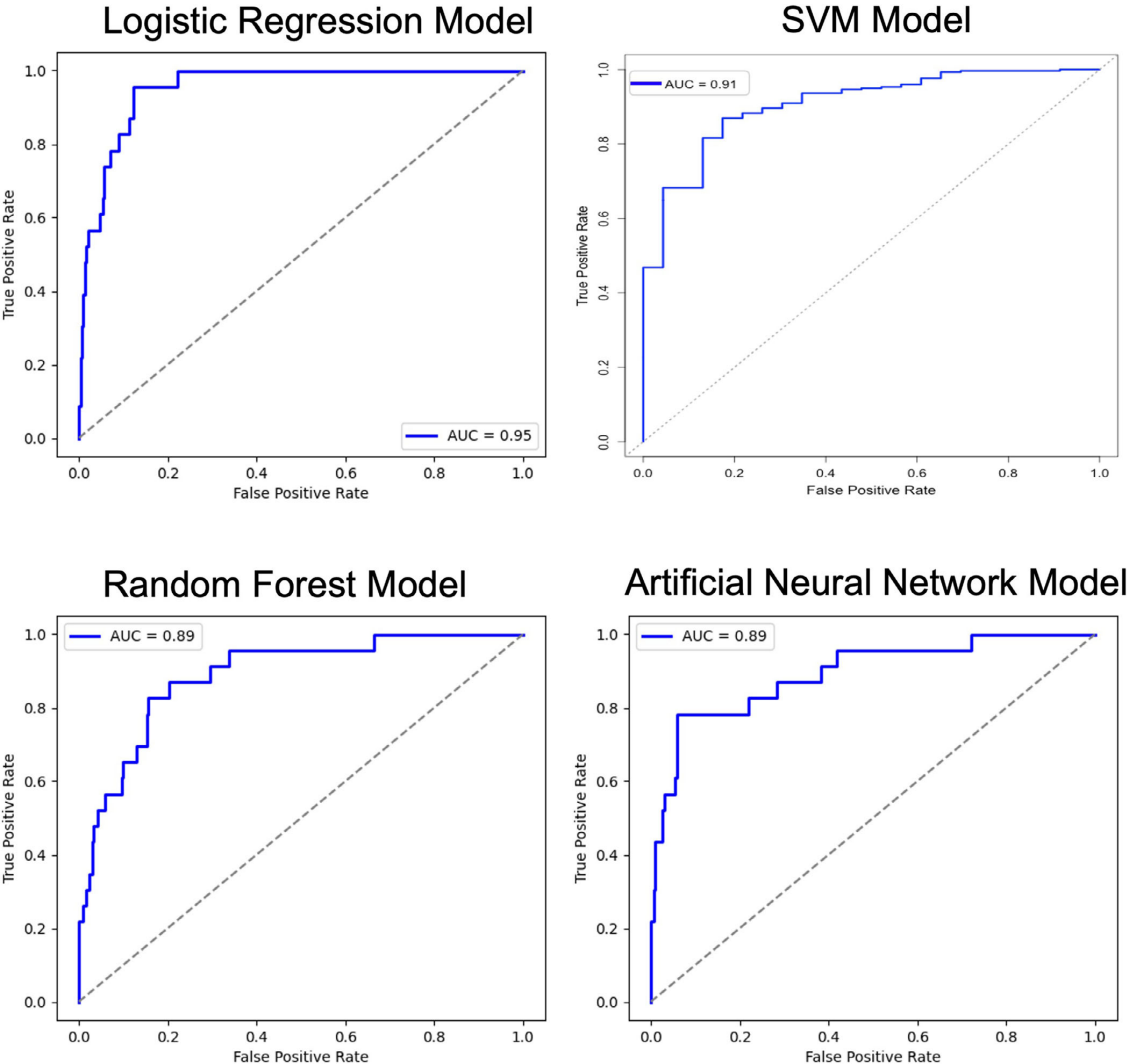
ROC curves for each classification model. Top left panel: logistic regression model, top right panel: SVM model, bottom left panel: random forest model, bottom right panel: neural network model. AUC, area under the curve; ROC, receiver operating characteristic; SVM, support vector machine.

**FIGURE 4:**
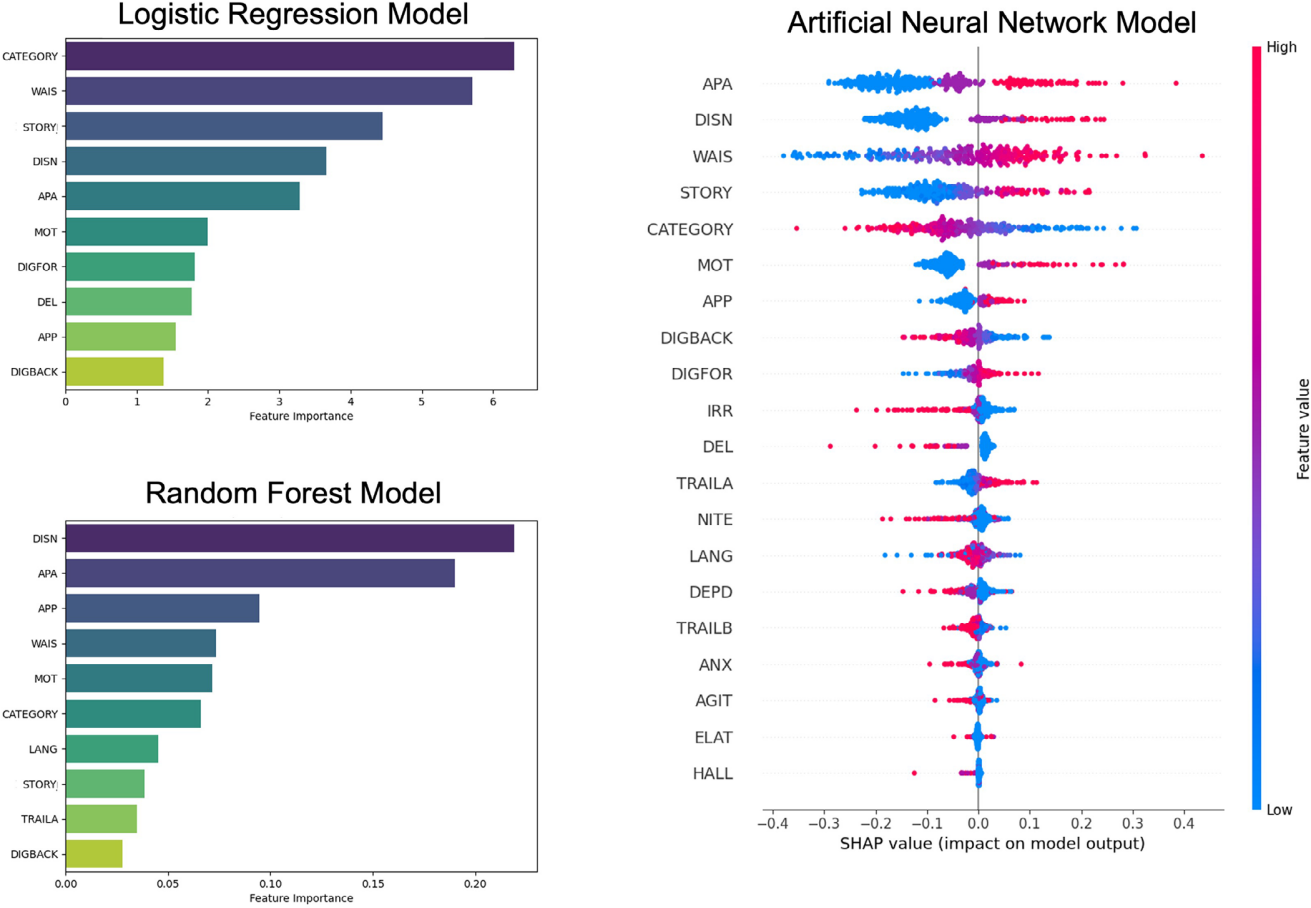
Important features in the three models. Important features in the three classification models where feature extraction is possible. *Y*‐axes refer to specific variables in the model and are ordered from most to least important based on each model's feature importance metric. Logistic regression (top left panel): *x*‐axis represents logistic regression coefficients, and higher coefficients indicate greater feature importance in predicting classification outcome. Random forest (bottom left panel): *x*‐axis represents feature importance scores calculated based on Gini impurity criterion; higher values indicate features that reduce impurity (e.g., are more important). Artificial neural network (right panel): *x*‐axis represents SHAP values for each feature, indicating impact each feature has on model's prediction; SHAP value magnitude corresponds to strength of feature's importance on model's outcome. AGIT, agitation/aggression; ANX, anxiety; APA, apathy/indifference; APP, appetite/eating problem; CATEGORY, category fluency; DEL, delusions; DEPD, depression/dysphoria; DIGBACK, Digit span/Number Span Test backward; DIGFOR, Digit span/Number Span Test forward; DISN, disinhibition; ELAT, elation/euphoria; HALL, hallucinations; IRR, irritability/lability; LANG, Boston Naming Test/Multilingual Naming Test; MOT, motor disturbance; NITE, nighttime behaviors; SHAP, Shapley Additive exPlanation; STORY, Logical Memory/Craft Story‐21 paraphrase score; TRAILA, Trail Making Test Part A; TRAILB, Trail Making Test Part B; WAIS, WAIS‐R Digit Symbol.

All 20 input features were min‐max normalized. SMOTE and class weights (AD × 1, bvFTD × 2) were applied to the training data prior to analysis. Alternate approaches to treat class imbalance (e.g., ADASYN) were applied to the training data, but the model performed best with SMOTE. A LR was tuned using the “lbfgs” solver with l2 regularization and 1000 iterations. The threshold was set to 0.6 in the test data model to balance sensitivity and specificity. The model achieved a classification accuracy of 88% (sensitivity = 91%, specificity = 88%, F1 = 52%, two false negatives) (Table 4) with good discrimination between groups (AUC = 0.95) (Figure 3). Category fluency, DSST, story memory, disinhibition, and apathy were the most important features for discriminating autopsy groups (Figure 4).

**TABLE 4:** Classification results from the four models.

Model	Sensitivity	Specificity	Accuracy	F1	AUC	PPV	NPV
Logistic regression	0.91	0.88	0.88	0.52	0.95	0.36	0.99
SVM	0.83	0.87	0.87	0.47	0.91	0.33	0.98
Random forest	0.83	0.80	0.80	0.37	0.89	0.24	0.98
ANN	0.78	0.91	0.90	0.53	0.89	0.40	0.98

*Notes*: Diagnosis was coded such that bvFTD was the positive class and AD was the negative class. Results represent metrics based on the first imputed dataset. Optimal models are reported.

Abbreviations: ANN, artificial neural network; AUC, area under the receiver operating characteristic curve; F1, harmonic mean of precision and recall; NPV, negative predictive value; PPV, positive predictive value; SVM, support vector machine.

#### 3.4.2 SVM

SVMs were trained to classify autopsy diagnosis based on the 12 NPI‐Q items and eight neuropsychological subtests. SMOTE was applied to the training data, but the model performed better without SMOTE and is reported as such here. Both linear and non‐linear kernels were applied, but the linear kernel had better performance and is therefore reported. All input features were scaled (*M* = 0, *SD* = 1) prior to analysis. Inverse weighting was applied to account for class imbalance, and the cost parameter (“C”) was set to 30 (higher values penalize false negatives) to balance misclassifications and overfitting. The model achieved a classification accuracy of 87% (sensitivity = 83%, specificity = 87%, F1 = 47%, four false negatives) (Table 4) with good discrimination between groups (AUC = 0.91) (Figure 3).

#### 3.4.3 Random forest

SMOTE was applied to the training data, which combines oversampling of the minority class (e.g., bvFTD) and undersampling of the majority class (e.g., AD) to achieve better model performance in the case of class imbalance.^50^ ADASYN was also applied to the data, but the model performed best with SMOTE. Class weights were also applied (AD × 1, bvFTD × 9) in addition to SMOTE to bias the model toward the minority class. Feature scaling through normalization or standardization did not improve the model, so the raw data were used in the final model. The optimal RF model was trained using 500 trees, a maximum tree depth of 8, a minimum leaf size of 5, and a minimum of 10 samples required to split a node. When validating the model, the threshold was set to 0.3 (vs default of 0.5) to maximize sensitivity (e.g., true positive bvFTD diagnosis) without sacrificing specificity and accuracy. The model achieved a classification accuracy of 80% (sensitivity = 83%, specificity = 80%, F1 = 37%, four false negatives) (Table 4) with good discrimination between classes (AUC = 0.89) (Figure 3). Disinhibition and apathy/indifference were the top two most important features for discriminating autopsy groups (Figure 4).

#### 3.4.4 Artificial neural networks

All input features were min‐max normalized prior to analysis. SMOTE and class weights (AD × 1, bvFTD × 1.1) were applied to the training data prior to analysis. Similar to the preceding models, ADASYN was also applied to the data, but the model performed best with SMOTE. Training was conducted over 75 epochs. The optimal ANN model consisted of one input layer (128 neurons), four hidden layers (128, 64, 32, and 16 neurons in each layer, respectively), and one output layer (one neuron). The ReLu activation function was applied to the input layer and hidden layers, and the sigmoid activation function was applied to the output layer. A threshold of 0.3 (vs default of 0.5) was applied to maximize sensitivity. This model achieved a classification accuracy of 90% (sensitivity = 78%, specificity = 91%, F1 = 53%, five false negatives) (Table 4) with good discrimination between classes (AUC = 0.89) (Figure 3). Apathy/indifference, disinhibition, and DSST were the top three most important features for discriminating groups (Figure 4).

### 3.5 Post hoc comparison of misclassified and accurately classified groups

We examined the clinical profile of misclassified bvFTD participants (i.e., false negative classifications) in each of the models (see Figure S1 for confusion matrices of each model). Overall, misclassified bvFTD participants tended to have lower neuropsychiatric symptom severity and worse performance on story memory relative to correctly classified bvFTD participants. The majority of misclassified bvFTD participants also endorsed mild to moderate anxiety, which was the most frequently endorsed symptom among those with AD. However, we are unable to determine whether the same participants were misclassified across models, so these findings should be interpreted with caution.

## 4 DISCUSSION

Differential diagnosis of bvFTD and AD based on clinical data is challenging due to significant overlap of symptom profiles, particularly at initial clinic visit.^3^, ^4^ We employed four robust ML techniques to classify AD and bvFTD based on initial visit neuropsychiatric symptom and neuropsychological test data. We found that a combination of neuropsychological and neuropsychiatric data collected at the initial visit was able to classify groups with high sensitivity and specificity. Apathy/indifference, disinhibition, and DSST were important clinical features for group classification across models, pointing to potential clinical diagnostic markers. We demonstrate that ML methods can complement clinical assessment by uncovering complex clinical patterns that may be difficult to detect through comparison of individual symptoms. ML can also identify accessible features that can help with differential diagnosis of AD and bvFTD, highlighting its potential for advancing clinical care.

### 4.1 Initial visit characteristics

The bvFTD sample was younger and had a higher rate of clinically assigned dementia compared to the AD sample, which is consistent with initial clinical presentation for these groups.^51^ NPI‐Q scores were higher for the bvFTD group compared to the AD group, reflecting greater behavioral and personality disturbances typical of bvFTD.^52^ Anxiety was the most frequently endorsed symptom among AD participants, while apathy/indifference and disinhibition were the most common in bvFTD.^5^, ^53^ Aspects of cognitive performance followed expected patterns for the two groups (e.g., AD performing worse than bvFTD on story memory),^54^ while other findings were less intuitive, such as bvFTD outperforming AD on executive functioning measures, though global cognition did not differ significantly between groups.

### 4.2 Classification of AD and bvFTD

All four of the ML models reliably classified AD and bvFTD groups with high discriminatory ability (AUC = 0.89 to 0.95), either matching or surpassing previously reported ML classification results using clinical data.^4^, ^14^, ^15^, ^16^, ^55^ F1 values were suboptimal due in part to the low bvFTD sample size; however, models consistently yielded few false negatives (i.e., misclassified bvFTD participants), which is notable given the difficulty of differential diagnosis in clinical settings.^5^, ^56^ Neuroimaging methods or biomarker methods have also shown high classification accuracy,^57^, ^58^ though these methods are less accessible.^2^, ^59^ Overall, the present findings highlight the reliability and accuracy of the combination of neuropsychological and neuropsychiatric symptoms for differential diagnosis of AD and bvFTD.

### 4.3 Identification of potential disease markers

Models consistently identified apathy, disinhibition, and DSST as important features for classification. Discrepancies between models may be due to the different metrics used for each model (e.g., Gini impurity for RF, SHAP for ANN). Despite these discrepancies, the consistency across models increases confidence in the ability of these clinical features to accurately classify groups.

#### 4.3.1 Apathy and disinhibition

Apathy and disinhibition were influential in the accurate classification of AD and bvFTD. Though not directly measured in our study, our neuropsychiatric findings support previous research implicating social cognition measures as important features for classification of these two groups.^55^ Apathy and disinhibition are considered hallmark features of bvFTD,^7^, ^10^, ^53^, ^60^ though apathy is also common in AD. Apathy is a complex, multifaceted neuropsychiatric presentation that can occur alongside other conditions (e.g., depression) or as an independent syndrome. Apathy manifests as reduced goal‐directed activity across behavioral (e.g., less initiation), cognitive (e.g., less interest in making decisions), emotional (e.g., reduced empathy), or social (e.g., less interest in socialization) contexts.^61^, ^62^ Additionally, apathy has negative implications for daily functioning,^63^ caregiver distress,^64^ and conversion from MCI to dementia.^65^ Disinhibition is similarly disruptive. Patients exhibit socially inappropriate behavior, loss of decorum, and impulsive or careless actions,^7^, ^66^ often leading to high caregiver distress.^52^, ^60^ While less common in AD, disinhibition was associated with increased likelihood of additional neuropsychiatric symptoms, highlighting the importance of assessing for this symptom in clinical settings.^67^ In summary, assessment of apathy and disinhibition during initial clinic visit may be critical due to their role in differential diagnosis of AD and bvFTD.

#### 4.3.2 Neuropsychological features for accurate classification

Certain neuropsychological subtests emerged as important features for accurate classification. For example, the DSST is a widely used neuropsychological test that measures a range of cognitive constructs including processing speed, visual scanning and attention, visuomotor functioning, working memory, and aspects of executive functioning.^68^ Performance on the DSST is also correlated with daily functioning^69^ and is a sensitive measure to detect unsafe driving among older adults.^68^ Category fluency and story memory were also important features identified by the LR model. As expected, story memory performance drastically differed between AD and bvFTD, though it was not as important for classification compared to other clinical features. Compared to the AD sample, the bvFTD sample had better performance on DSST and worse performance on category fluency. Group differences in neuropsychological performance may be sample‐specific. Despite higher rates of dementia in our bvFTD sample, global cognition was similar between the groups, highlighting the importance of examining a combination of features.

Overall, our findings support the value of neuropsychiatric symptom assessment and neuropsychological tests for discrimination of AD and bvFTD. Importantly, these tools are brief, easy to administer, and accessible and may represent promising clinical markers for differential diagnosis.

### 4.4 Characteristics of misclassified bvFTD

The ML models were able to accurately classify the majority of bvFTD participants, with two to five false negatives in each model. When comparing the accurately classified bvFTD participants with the misclassified bvFTD participants (i.e., false negatives), we observed that misclassified participants had lower overall neuropsychiatric symptom severity, higher rates of anxiety, and worse performance on story memory. This supports previous findings that showed misdiagnosed bvFTD (i.e., pathologically confirmed FTLD but diagnosed as AD) exhibited an AD phenotypic profile, characterized by fewer neuropsychiatric symptoms, older age, and worse memory performance.^9^ However, clinical characteristics of our misclassified group were variable, and future research is needed to determine appropriate classification strategies for these atypical cases. Patients with atypical clinical presentations may be the best candidates for whom biomarker testing should be prioritized, where available.

### 4.5 Strengths, limitations, and future directions

The NACC UDS and Neuropathology Data Sets provide a large, well‐phenotyped, and autopsy‐confirmed sample, which increases generalizability and reliability of our findings. NACC uniquely includes longitudinal measurement of these factors, which has enabled ground‐breaking research that advances precise detection and facilitates differential diagnosis of ADRD. We tested four robust ML models capable of handling the complexity and non‐linearity of clinical data, improving classification precision and accuracy.^39^ However, several limitations are worth noting.

Though our imbalanced samples represent current base rates of AD and bvFTD, imbalanced samples can worsen model performance. While SMOTE is a standard method for addressing class imbalance, it can generate synthetic data based on atypical cases in the training set, potentially impacting the generalizability of findings. Therefore, analyses should be replicated in larger, balanced samples to ensure the reliability of results. The primary limiting factor for achieving balanced samples is the availability of *post mortem* neuropathology data. NACC and the ADRC program remain committed to identifying and sharing this source of participant data; these initiatives are critical for research. Translating research into practice is essential for advancing patient care and discovering new disease markers, but the “black‐box” nature of ML can limit the translational impact of findings.^13^

Despite its importance in differential diagnosis, evaluation of social functioning is not typically included in a standard clinical battery^70^ and was, therefore, not accessible in this dataset. Thus, while previous research highlighted the importance of social functioning in discriminating AD and bvFTD,^55^, ^71^, ^72^ we were unable to verify this in the present study. Aspects of our findings (e.g., importance of apathy and disinhibition), however, emphasize the importance of the assessment of social functioning. Future work is needed to identify and incorporate comprehensive measures of social functioning into the clinical evaluation of bvFTD and AD. Additionally, examining the use of clinical symptoms for classification of bvFTD and primary psychiatric conditions may be useful, given the high rates of misdiagnosis between bvFTD and primary psychiatric conditions.^73^, ^74^ Finally, our sample was predominantly non‐Hispanic white and not representative of the larger patient population. It is especially important to replicate this study with a more diverse sample, as racial and ethnic minorities have a higher proportion of missed or delayed dementia diagnosis.^74^

### 4.6 Summary

This study confirms the effectiveness of clinical measures for the classification of AD and bvFTD. We addressed critical gaps in the literature by leveraging pathologically verified samples, incorporating both neuropsychiatric and neuropsychological data for classification, and systematically evaluating multiple ML models. Findings emphasize the importance of neuropsychological and neuropsychiatric assessment during the initial clinic visit. We also identify promising clinical markers for the differential diagnosis of AD and bvFTD. The use of accessible, clinical features enhances the potential for broad application.

## CONFLICT OF INTEREST STATEMENT

J.L.C. has provided consultation to Acadia, Acumen, ALZpath, Annovis, Aprinoia, Artery, Biogen, Biohaven, BioXcel, Bristol‐Myers Squib, Eisai, Fosun, GAP Foundation, Green Valley, Janssen, Karuna, Kinoxis, Lighthouse, Lilly, Lundbeck, LSP/eqt, Mangrove Therapeutics, Merck, MoCA Cognition, New Amsterdam, Novo Nordisk, Optoceutics, Otsuka, Oxford Brain Diagnostics, Praxis, Prothena, ReMYND, Roche, Scottish Brain Sciences, Signant Health, Simcere, sinaptica, T‐Neuro, TrueBinding, and Vaxxinity pharmaceutical, assessment, and investment companies. J.L.C. owns the copyright of the Neuropsychiatric Inventory. The remaining authors (G.J.G., J.F., S.M., S.E.J.) have no conflicts to disclose. Author disclosures are available in the Supporting Information.

## CONSENT STATEMENT

Participants and study partners enrolled at each ADRC provided written consent as part of the Institutional Review Board‐approved protocol at that site. This consent covers both the data collection procedures required by the respective center as well as the inclusion of the participants’ data in the larger NACC UDS database.

## Supporting information



Supporting Information

Supporting Information
