# Supplementary material for: Classification of AD and bvFTD using neuropsychological and neuropsychiatric variables: a machine learning study
Source: Alzheimers Dement. 2025 Oct 21;21(10):e70782. doi: 10.1002/alz.70782 (PMC12538632; doi:10.1002/alz.70782)
Supplement: Supplementary file 1 — Supporting Information [file ALZ-21-e70782-s002.docx]

**Supplementary Materials**

Supplementary Table 1. Frequency of Neuropsychiatric Symptom Severity Ratings by Autopsy Group

|  | **No Symptoms (0)** | **Mild (1)** | **Moderate (2)** | **Severe (3)** |
| --- | --- | --- | --- | --- |
| Delusions |  |  |  |  |
| AD | 87.57% | 7.17% | 4.17% | 1.09% |
| bvFTD | 88.60% | 4.39% | 5.26% | 1.75% |
| Hallucinations |  |  |  |  |
| AD | 95.15% | 3.55% | 1.09% | 0.21% |
| bvFTD | 94.74% | 3.51% | 0.88% | 0.88% |
| Agitation/Aggression^*^ |  |  |  |  |
| AD | 69.95% | 18.17% | 9.70% | 2.19% |
| bvFTD | 54.39% | 23.68% | 16.67% | 5.26% |
| Depression/Dysphoria |  |  |  |  |
| AD | 62.57% | 25.14% | 10.79% | 1.50% |
| bvFTD | 67.54% | 15.79% | 15.79% | 0.88% |
| Anxiety^*^ |  |  |  |  |
| AD | 59.77% | 24.73% | 12.91% | 2.60% |
| bvFTD | 55.26% | 20.18% | 18.42% | 6.14% |
| Elation/Euphoria^**^ |  |  |  |  |
| AD | 95.69% | 2.94% | 1.23% | 0.14% |
| bvFTD | 77.19% | 7.02% | 12.28% | 3.51% |
| Apathy/Indifference^**†^ |  |  |  |  |
| AD | 62.09% | 22.00% | 12.91% | 3.01% |
| bvFTD | 14.91% | 21.93% | 36.84% | 26.32% |
| Disinhibition^**†^ |  |  |  |  |
| AD | 80.26% | 12.84% | 5.06% | 1.84% |
| bvFTD | 28.95% | 27.19% | 26.32% | 17.54% |
| Irritability/Lability^**^ |  |  |  |  |
| AD | 62.68% | 23.38% | 11.35% | 2.60% |
| bvFTD | 51.75% | 21.93% | 16.67% | 9.65% |
| Motor Disturbance^**^ |  |  |  |  |
| AD | 82.84% | 9.98% | 5.47% | 1.71% |
| bvFTD | 40.35% | 15.79% | 28.95% | 14.91% |
| Nighttime Behaviors^**^ |  |  |  |  |
| AD | 74.90% | 14.09% | 9.03% | 1.98% |
| bvFTD | 58.77% | 18.42% | 19.30% | 3.51% |
| Appetite/Eating Problems^**^ |  |  |  |  |
| AD | 75.03% | 14.64% | 8.76% | 1.57% |
| bvFTD | 35.09% | 15.79% | 35.09% | 14.04% |

*Note.* Aggregate neuropsychiatric symptom ratings derived from the NPI-Q. Item-level ratings: no symptoms = 0, mild = 1, moderate = 2, severe = 3. AD = Alzheimer’s disease. bvFTD = behavioral variant frontotemporal dementia. ^*^Groups differed at *p* < .05.^**^Groups differed at *p* < .001. ^†^ Denotes important feature in the classification models.

Supplementary Figure 1. Confusion Matrices for Each Model


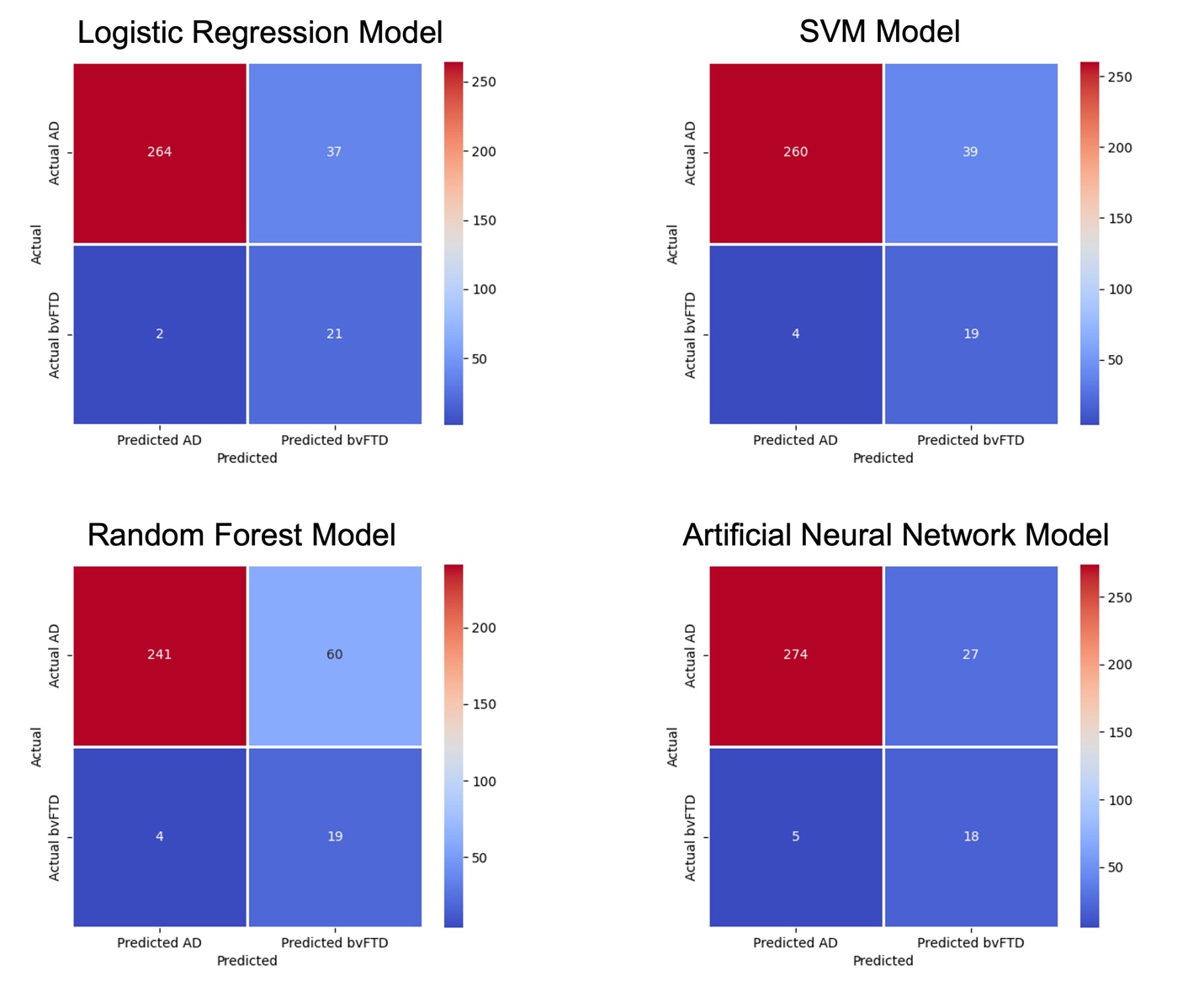


*Note.*  Confusion matrix for each classification model. Colors for each square correspond with the color assignments based on the number of participants in each square (color thermometer). Top left panel: logistic regression model, top right panel: SVM model, bottom left panel: random forest model, bottom right panel: neural network model. AD = Alzheimer’s disease. bvFTD = behavioral variant frontotemporal dementia. SVM = support vector machines.
